# Use of an IL-1 Inhibitor for Refractory Steroid-Dependent Lupus Pericarditis With Constrictive Physiology

**DOI:** 10.1016/j.jaccas.2025.105348

**Published:** 2025-09-09

**Authors:** Patrick Creechan, Megan Lockwood, Brittany N. Weber, Allan Klein, Syed W. Haider

**Affiliations:** a Pericardial Disease Program, MedStar Heart and Vascular Institute, Washington, District of Columbia, USA; b Division of Rheumatology, MedStar Georgetown University Hospital, Washington, District of Columbia, USA; c Division of Cardiovascular Imaging, Brigham and Women's Hospital, Boston, Massachusetts, USA; d Center for the Diagnosis and Treatment of Pericardial Diseases, Section of Cardiovascular Imaging, Robert and Suzanne Tomsich Department of Cardiovascular Medicine, Sydell and Arnold Miller Family Heart, Vascular and Thoracic Institute, Cleveland Clinic, Cleveland, Ohio, USA

**Keywords:** cardiac magnetic resonance, constrictive pericarditis, recurrent pericarditis, rilonacept, steroid-sparing therapy, systemic lupus erythematosus

## Abstract

**Background:**

Pericardial involvement is common in systemic lupus erythematosus (SLE) and can lead to recurrent episodes. B cell–targeted therapies are commonly used in the treatment of SLE pericarditis. The management of recurrent lupus pericarditis refractory to B cell–targeted therapy remains challenging.

**Case Summary:**

A 33-year-old woman with SLE developed steroid-dependent, recurrent pericarditis with large effusions requiring 2 pericardial windows. Despite corticosteroids, colchicine, hydroxychloroquine, and the B cell–targeted therapy belimumab, she had persistent symptomatic recurrences with features of evolving constrictive physiology on cardiac magnetic resonance. Transition to rilonacept led to rapid symptom resolution, steroid discontinuation, and cardiac magnetic resonance–documented reversal of pericardial inflammation and constrictive physiology.

**Discussion:**

This case illustrates that interleukin-1 inhibition with rilonacept can be an effective steroid-sparing strategy for refractory lupus pericarditis after failure of traditional B cell–targeted therapy.

**Take-Home Messages:**

Lupus pericarditis can be steroid dependent and refractory to standard SLE immunosuppression, including B cell–directed biologics. Interleukin-1 inhibition offers a potential therapeutic strategy for breaking the autoinflammatory cycle that can sustain recurrent pericarditis.

## Visual Summary

**Figure:**
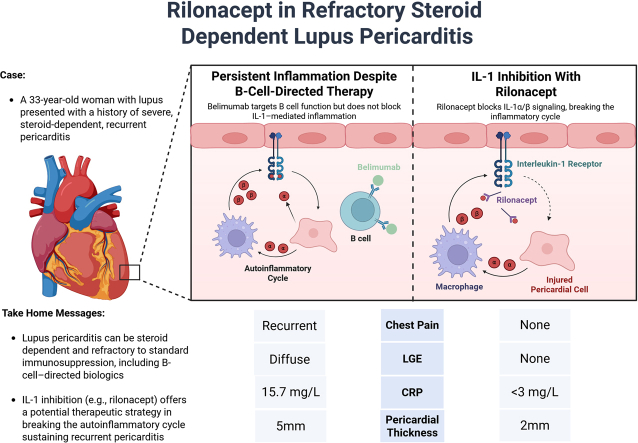

**Visual Summary:**
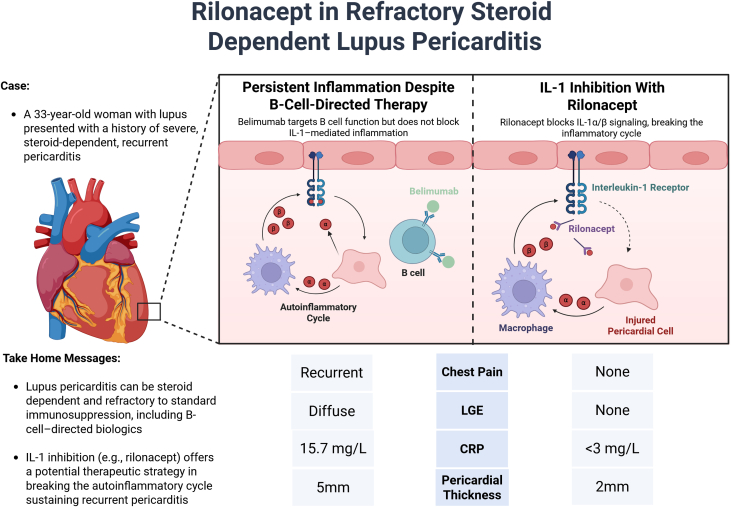
Contrasting Effects of B Cell–Directed Therapy vs IL-1 Inhibition in a Patient With Recurrent Lupus Pericarditis On the left, belimumab fails to interrupt interleukin (IL)-1 –mediated autoinflammatory signaling, allowing persistent inflammation despite systemic lupus control. On the right, rilonacept acts as an IL-1α/IL-1β cytokine trap, blocking receptor engagement and disrupting the inflammatory cycle. Clinical response to rilonacept included resolution of chest pain, normalization of inflammatory markers, and reversal of pericardial late gadolinium enhancement and thickening on cardiac magnetic resonance. Figure created in BioRender. CRP = C-reactive protein; LGE = late gadolinium enhancement.

## History of Presentation

**Figure 1:**
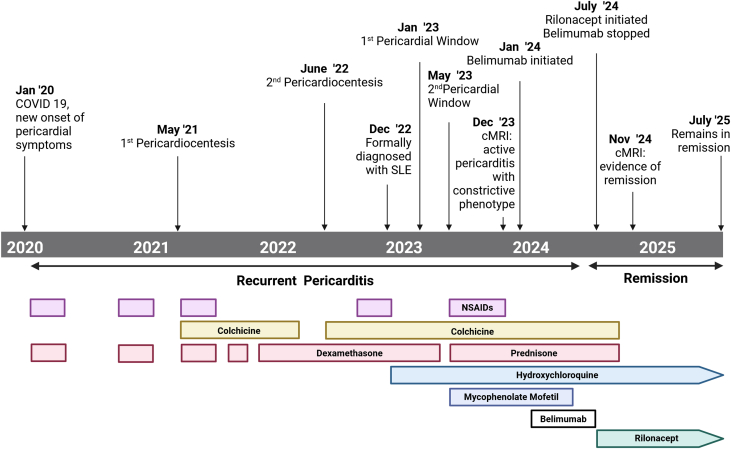
Timeline of Events This timeline illustrates the progression of recurrent pericarditis, detailing the chronology of procedural and pharmacologic interventions. Created with BioRender.com. cMRI = cardiac magnetic resonance imaging; NSAID = nonsteroidal anti-inflammatory drug; SLE = systemic lupus erythematosus.

A 33-year-old woman with systemic lupus erythematosus (SLE) presented with a history of severe, steroid-dependent, recurrent pericarditis (Figure 1). She first presented with chest pain and shortness of breath following a coronavirus disease 2019 infection 3 years ago. She was diagnosed with acute pericarditis and was treated with colchicine. In early 2021, she had a persistent pericardial effusion, which led to tamponade physiology requiring emergent pericardiocentesis. Despite nonsteroidal anti-inflammatory drugs, colchicine, and corticosteroids, she had repeated symptomatic recurrences with large effusions. She underwent a first pericardial window procedure in January 2021. Persistent effusions and clinical recurrences led to a second, larger pericardial window in May 2021.

**Take-Home Messages**

• Lupus pericarditis can be steroid dependent and refractory to standard immunosuppression, including B cell–directed biologics.
• IL-1 inhibition (eg, rilonacept) offers a potential therapeutic strategy by interrupting the autoinflammatory cycle sustaining recurrent pericarditis.

Pericardial fluid analyses were sterile, exudative, and cytologically benign. Biopsies showed chronic fibrinous inflammation without granulomas or malignancy. Infectious evaluations, including tuberculosis screening, blood cultures, and viral polymerase chain reaction, were negative.

During this period, SLE, based on positive antinuclear antibody titer (1:160, homogeneous pattern), anti–double-stranded DNA (dsDNA) antibodies (40 IU/mL), low complement levels (C3, 85 mg/dL; C4, 12 mg/dL), and inflammatory arthritis, supported by anti-Ro60 antibodies (23 U/mL), were diagnosed. There was no evidence of lupus nephritis, central nervous system involvement, or other major organ disease. Her initial SLE treatment regimen was hydroxychloroquine 200 mg twice daily and mycophenolate mofetil 1,000 mg twice per day. She continued dexamethasone 1 to 4 mg daily (subsequently transitioned to prednisone) and colchicine 0.6 mg twice daily.

Despite this regimen, she remained highly corticosteroid dependent for pericarditis control. Pericarditis symptoms consistently flared whenever prednisone was tapered to <15 mg daily. Notably, her systemic lupus manifestations and serologic markers of lupus activity remained clinically quiescent during these episodes, suggesting isolated pericardial disease activity. Inflammatory markers, including high-sensitivity C-reactive protein (CRP) and erythrocyte sedimentation rate (ESR), rose intermittently with recurrent episodes with peak values of high-sensitivity CRP 15.7 mg/L and ESR 39 mm/h, with lower values often presumed to be masked by ongoing steroid therapy.

Mycophenolate was discontinued owing to lack of response. Belimumab, a monoclonal antibody targeting the B-lymphocyte stimulator (BLyS) was added in January 2024 to improve lupus control and reduce steroid burden. Over the course of 6 months, belimumab failed to prevent symptomatic recurrences or allow meaningful prednisone tapering. However, her complement levels and dsDNA antibodies normalized.

## Investigation

**Figure 2:**
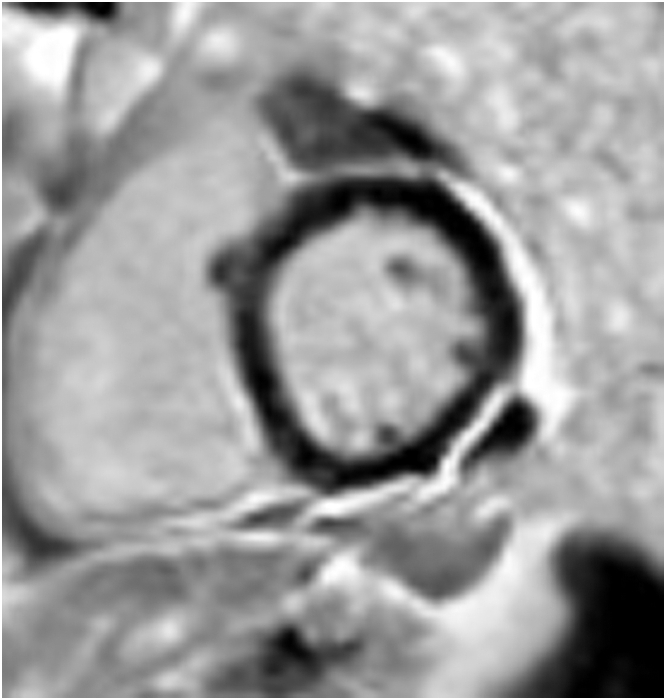
Pre-Rilonacept Cardiac Magnetic Resonance Cardiac magnetic resonance showing a diffusely thickened pericardium (4-5 mm) and circumferential late gadolinium enhancement.

Echocardiography demonstrated preserved biventricular function with abnormal interventricular septal motion (septal bounce) and >25% respiratory variation in mitral inflow velocities, suggesting evolving constrictive physiology. Cardiac magnetic resonance (CMR) (Figure 2) demonstrated diffuse pericardial thickening (4-5 mm) with circumferential late gadolinium enhancement (LGE), consistent with active pericardial inflammation. Real-time cine demonstrated diastolic septal shift with exaggerated respiratory variation, confirming inflammatory constrictive physiology.

## Management

Given persistent, steroid-dependent pericarditis unresponsive to guideline-directed therapy and failure of B cell–targeted biologic therapy (belimumab), a multidisciplinary cardiology and rheumatology team decision was made in July 2024 to transition to interleukin (IL)-1 inhibition with rilonacept and discontinue both mycophenolate and belimumab. Rilonacept was initiated with a 320-mg subcutaneous loading dose, followed by 160-mg weekly maintenance dosing. Hydroxychloroquine was continued. Colchicine and prednisone were discontinued following structured tapers.

## Outcome and Follow-Up

**Figure 3:**
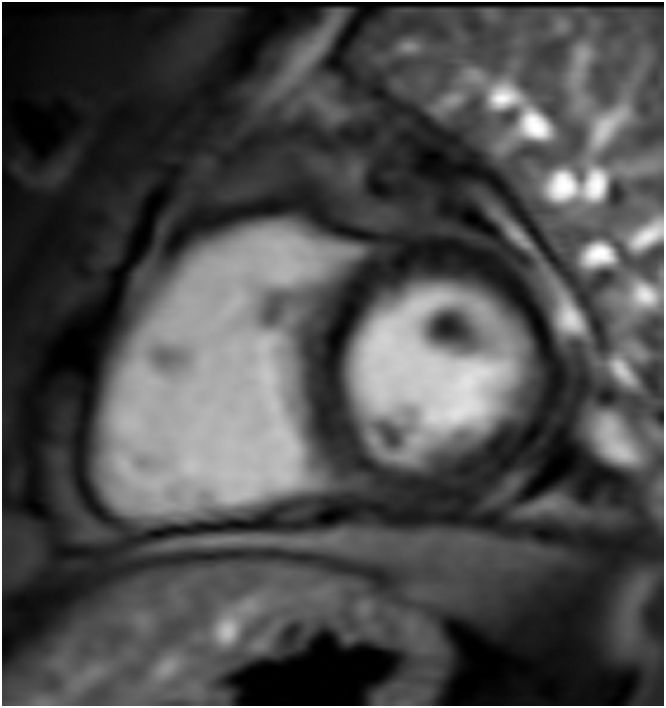
Post-Rilonacept Cardiac Magnetic Resonance Cardiac magnetic resonance showing normalization of pericardial thickness to 2 mm and complete resolution of pericardial late gadolinium enhancement.

The patient experienced marked improvement within 3 weeks of rilonacept initiation, with complete resolution of chest pain. She tolerated a 2-month prednisone taper without recurrence of symptoms. Follow-up CMR demonstrated complete resolution of pericardial LGE, normalization of septal motion, and reduction of pericardial thickness to 2 mm (Figure 3).

At 12 months after initiation of rilonacept, the patient remains asymptomatic, without the use of any corticosteroids, with normal inflammatory markers (CRP <1 mg/L, ESR 2-7 mm/h). She continues weekly rilonacept and hydroxychloroquine without recurrence of pericardial effusion and has had no infections or adverse effects apart from mild injection site reactions, managed conservatively with topical steroids. The patient remains under regular follow-up with the cardiology and rheumatology teams.

## Discussion

Pericarditis is a common clinical manifestation of SLE, reported in up to 20% to 30% of patients, although many cases are subclinical.^1^^,^^2^ Most patients respond to escalation of lupus therapy with some requiring additional nonsteroidal anti-inflammatory drugs, colchicine, and corticosteroids. However, a subset develop recurrent or refractory pericarditis that is steroid dependent and difficult to manage.^3^ High-dose or prolonged steroid use itself is a known risk factor for recurrence.^2^^,^^4^

In this patient, early pericarditis episodes preceded a formal SLE diagnosis, but occurred in the context of evolving systemic autoimmunity, with positive antinuclear antibody, anti-dsDNA antibodies, and low complement levels. Her SLE manifestations and serologic activity were otherwise mild, underscoring that pericardial disease may dominate the clinical course in some patients. Despite background therapy with hydroxychloroquine and a trial of belimumab, she remained highly steroid dependent for pericarditis resulting in multiple hospitalizations and emergency department visits.

This case highlights an important distinction in the pathophysiology of lupus pericarditis: though traditionally a B cell–mediated autoimmune process, some cases exhibit a recurrent, steroid-dependent course more consistent with autoinflammatory disease. In such patients, autoimmune processes sustain chronic inflammation alongside an autoinflammatory IL-1–mediated feedback loop.^5^ These patients may not respond as well to B cell–targeted therapies. The RHAPSODY trial showed that rilonacept (an IL-1α/β cytokine trap) dramatically reduced recurrence risk in idiopathic recurrent pericarditis.^6^ Data for SLE-associated pericarditis remain limited but are increasing. Case reports and small series have documented successful use of anakinra^7^ and rilonacept^5^ in refractory lupus pericarditis. Our case contributes to this evolving evidence base, demonstrating that rilonacept can achieve clinical remission and steroid discontinuation even after failure of B cell–directed therapy, steroids, and colchicine. Recognition of an autoinflammatory phenotype in select patients with lupus pericarditis may allow for more targeted and effective treatment.

Advanced imaging played a central role in guiding treatment decisions in this case. CMR revealed circumferential LGE of the pericardium with thickening and real-time diastolic septal shift—definitive markers of active inflammatory constrictive physiology. Recognition of this potentially reversible state was critical in selecting targeted anti-inflammatory therapy. Importantly, timely initiation appeared to prevent progression to irreversible or “burnt out” constrictive physiology, as evidenced by CMR-documented resolution of LGE and normalization of diastolic septal motion.^8^

This case also underscores the need for early, multidisciplinary collaboration between cardiology and rheumatology in managing complex lupus pericarditis.^9^ As highlighted by Brown and Kontzias,^10^ such collaboration enables appropriate imaging, diagnostic clarification of active inflammation vs fibrosis, and individualized therapeutic strategies to reduce steroid burden and avoid surgical pericardiectomy. In our patient, close coordination across specialties supported timely decision making, and ultimately contributed to a favorable outcome.

## Conclusions

This case demonstrates that IL-1 inhibition with rilonacept can achieve remission in steroid-dependent, recurrent lupus pericarditis after failure of B cell–targeted therapy. By interrupting the autoinflammatory cycle sustaining pericardial inflammation in this patient, rilonacept enabled steroid discontinuation and prevented progression to constrictive physiology. Clinicians should maintain a high index of suspicion for this mechanism in refractory cases and consider IL-1 blockade early as part of a multidisciplinary, steroid-sparing approach.

## Funding Support and Author Disclosures

Dr Haider serves on the advisory board for Kiniksa. Dr Klein has received research funding and has served on scientific advisory boards for 10.13039/100016492Kiniksa Pharmaceuticals, Cardiol Therapeutics, and Ventyx. Dr Weber has received grant support from the 10.13039/100000050National Heart, Lung, and Blood Institute (K23 HL159276-01, AHA 21CDA851511); she has received advisory panel/consultant fees from Novo Nordisk, Oruka, and BMS. All other authors have reported that they have no relationships relevant to the contents of this paper to disclose.
